# PathoPhenoDB, linking human pathogens to their phenotypes in support of infectious disease research

**DOI:** 10.1038/s41597-019-0090-x

**Published:** 2019-06-03

**Authors:** Şenay Kafkas, Marwa Abdelhakim, Yasmeen Hashish, Maxat Kulmanov, Marwa Abdellatif, Paul N. Schofield, Robert Hoehndorf

**Affiliations:** 1 Computer, Electrical and Mathematical Sciences & Engineering with Division, Computational Bioscience Research Center, King Abdullah University of Science and Technology, Thuwal, 23955 Saudi Arabia; 2 Department of Physiology, Development & Neuroscience, University of Cambridge, Downing Street, Cambridge, CB2 3EG United Kingdom

**Keywords:** Data integration, Infectious diseases

## Abstract

Understanding the relationship between the pathophysiology of infectious disease, the biology of the causative agent and the development of therapeutic and diagnostic approaches is dependent on the synthesis of a wide range of types of information. Provision of a comprehensive and integrated disease phenotype knowledgebase has the potential to provide novel and orthogonal sources of information for the understanding of infectious agent pathogenesis, and support for research on disease mechanisms. We have developed PathoPhenoDB, a database containing pathogen-to-phenotype associations. PathoPhenoDB relies on manual curation of pathogen-disease relations, on ontology-based text mining as well as manual curation to associate host disease phenotypes with infectious agents. Using Semantic Web technologies, PathoPhenoDB also links to knowledge about drug resistance mechanisms and drugs used in the treatment of infectious diseases. PathoPhenoDB is accessible at http://patho.phenomebrowser.net/, and the data are freely available through a public SPARQL endpoint.

## Abstract (summary)

**Table:** 

Design Type(s)	disease state design • disease analysis objective • data integration objective
Measurement Type(s)	association
Technology Type(s)	digital curation
Factor Type(s)	pathogen • phenotype
Sample Characteristic(s)	Homo sapiens

Machine-accessible metadata file describing the reported data (ISA-Tab format)

## Background & Summary

The 2016 Global burden of disease study estimated that infectious diseases as part of the communicable, maternal, nutritional and neonatal complex (CMNN) accounted for 19% of global mortality in 2016, and constitute the second most important cause of deaths globally^1^. They remain the top cause of mortality in most of the developing countries, mainly in Africa, at 56% in 2015. The annual infectious disease mortality in the world is reported as 10,598 (per 100,000 people) by the World Health Organization (WHO). Lower respiratory tract infections are the most likely cause of mortality due to infectious disease, followed by diarrhoeal diseases, tuberculosis and HIV/AIDS which were responsible for 3.2 million, 1.4 million, 1.4 million and 1.1 million deaths respectively in 2015 alone (http://who.int/en/). Infectious diseases have highly significant economic impact through morbidity and mortality, especially for the developing countries^2^. They affect multiple components of human development including income, health, education and productivity through lost life years, and cause devastating consequences worldwide.

Infectious diseases are caused by a wide range of organisms (viruses, bacteria, fungi, worms, protozoa) that are generally considered as pathogens. Antimicrobial, antihelminthic, antiprotozoal and antifungal drugs are often the first line therapy for infectious diseases. However, drug resistance accumulates over time due to selection of genetic changes in pathogen populations when they are exposed to therapeutic agents. It now becomes crucial to develop strategies that can identify a pathogen rapidly and determine successful treatment options based on functional information in the pathogen relevant to drug resistance mechanisms.

While functional information about pathogens and their interactions with hosts is increasingly becoming available on a molecular level through large-scale studies^3^, disease phenotypes observed in an infected patient are not only mediated through direct molecular interactions between pathogen and host but also through the immune response and physiological and patho-physiological processes affecting the entire host organism. Phenotypes observed in a patient provide a readout for all these processes and generally provide a proxy for the mechanism through which pathogens elicit their signs and symptoms^4^. While there is a wide range of phenotypes that are shared across multiple infectious diseases as a result of common immune system processes and immune response to pathogens, certain host-pathogen interactions may result in specific phenotypes through which pathogens can be broadly distinguished.

Phenotype-based computational analysis methods can uncover molecular mechanisms in Mendelian diseases^5^, and have been applied to the discovery of disease mechanisms from animal models^6^ and to the investigation of drug mechanisms and drug repurposing^7^. In the area of infectious disease, similar methods may be applicable, mainly to investigate mechanisms of virulence and pathogenicity. Application of phenotype-based methods requires matching phenotypes observed in a particular physiological or pathological state with the phenotypes known to be associated with pathogens^6,8^, and use of this information to reveal molecular mechanisms. Currently, there is no comprehensive database of pathogen-to-phenotype associations that can be used for this purpose.

We have developed PathoPhenoDB, a database of pathogen-to-phenotype associations intended to support infectious disease research. PathoPhenoDB is a database which relies on pathogen–disease associations curated manually from public resources and the scientific literature. We further expanded the pathogen–disease associations by complementary text-mined data^9^. PathoPhenoDB links pathogens to disease phenotypes based on manually-curated and text-mined disease–phenotype associations. Furthermore, PathoPhenoDB links pathogens to drugs^10^ that are known to treat infections by the pathogen, and further links pathogens to drug resistance genes and proteins^11^ as well as to the drugs against which these genes or proteins convey resistance so that the information in PathoPhenoDB can be utilized directly for research on drug resistance mechanisms. PathoPhenoDB is freely available on http://patho.phenomebrowser.net, and the data can be obtained through a public SPARQL endpoint.

## Methods

### Data collection and integration

We developed PathoPhenoDB by considering the FAIR data principles^12^. Our expert gathered manually at least one possible human pathogen for every of infectious disease class listed in the Human Disease Ontology (DO)^13^ from public resources. Therefore, we cover all of the infectious diseases from DO and intend to maintain this coverage with future extensions of DO. We gathered the pathogen–disease associations mainly from the Centers for Disease Control and Prevention (CDC) (https://www.cdc.gov/, 87%) as well as from Wikipedia (mainly from the list of infectious disease, https://en.wikipedia.org/wiki/List_of_infectious_diseases), the Disease Ontology, and literature in PubMed; we used the labels of the infectious disease for which we wanted to identify a causative pathogen as search terms.

We used lexical matching to map the gathered diseases to DO and pathogens to NCBI Taxonomy semi-automatically. In the cases where we could not find an exact match of a pathogen in the NCBI taxonomy, we mapped the pathogen to their parent class. For example, instead of assigning *Spirillium minus* to *Sodoku disease*, we assigned the higher taxon *Spirillium* to *Sodoku disease* due to *Spirillium minus* not being listed in the NCBI taxonomy.

Furthermore, we extracted pathogen–disease associations from all the Open Access full-text PMC articles (version:v.2017.12, size: 1.8 million) from Europe PMC^14^ by using an ontology-based text mining approach^9^, and we included the resulting association in PathoPhenoDB. Briefly, our system extracts pathogen–disease association from text in three steps. First, it annotates pathogen and disease names in text by using dictionaries compiled from NCBI Taxonomy and DO and propagates the annotations by using the hierarchical class structure defined in the ontologies. Second, it identifies pathogen–disease pairs based on their co-occurrences at the sentence level. Third, it applies a statistical analysis to filter the pairs based on their co-occurrence statistics and the Normalized Pointwise Mutual Information (NPMI)^15^ measure. We evaluated the performance of our system manually on 50 randomly selected pathogen–disease associations. Our system achieved a precision of 64%, a recall of 84% and an F-score of 73%^9^.

Here, we consider infectious disease phenotypes as those observable or measurable characteristics of a host which constitute the signs and symptoms of the pathogen-induced disease. We restrict the range of phenotypic characteristics we consider to the phenotypes that are represented by classes in the Human Phenotype Ontology (HPO)^16^ or the Mammalian Phenotype (MP) ontology^17^.

Disease phenotypes were extracted semi-automatically from class definitions in the Disease Ontology and automatically from PubMed abstracts by using ontology-based text mining^18^. Briefly, during our manual extraction process, we mapped the phenotypes to HPO and MP automatically where there is an exact match otherwise we mapped them manually. Overall, we were able to map 90% (293/324) of the phenotypes linked to the infectious diseases in DO to HPO and MP. For text mining the disease–phenotype associations, we first identified co-occurrences between disease names from DO and phenotype names from the Human Phenotype Ontology (HPO) (downloaded on 14 May 2018) and the Mammalian Phenotype Ontology (MP) (downloaded on 14 May 2018) in all PubMed abstracts (approximately 28 million). To integrate the HPO and MP ontologies we use the PhenomeNET ontology^6^. Secondly, we identified the disease–phenotype co-occurrences and calculated the strength of each co-occurrence based on an ontology-based normalized point-wise mutual information^18,19^. Thirdly, we selected a threshold for the most “useful” co-occurrences by evaluating the performance of our system in candidate disease gene prediction at different NPMI ranks. Our evaluation results show that the system achieves its best performance at the NPMI rank 12.

We extracted drug information from the SIDER database^10^ by resolving the cross-references between UMLS concept identifiers and DO identifiers. We gathered information about drug resistance from the Antibiotic Resistance Ontology (ARO)^11^. For this purpose, we matched the ARO accession and iterated through the ontology hierarchy using the subclass and *confers-resistance-to* relationships in ARO to retrieve the drugs. Using the Entrez API, we then identified the DNA accession that conveys drug resistance in the NCBI nucleotide database to retrieve the organism and its NCBI Taxonomy identifier. While, diseases are linked to pathogens, drugs, and phenotypes, the pathogens are linked to their drug resistance proteins, DNA accession and drugs to which they are resistant.

### Semantic similarity computation

We calculate the semantic similarities using Resnik’s semantic similarity measure^20^. We represent each pathogen *p* with a set containing all the possible phenotypes it can cause in its host (based on the data in PathoPhenoDB). We formulate a query by a set of phenotypes and rank pathogens based on their similarity scores to the query. Resnik’s semantic similarity measurement is based on information content of a class *c* which we define as the negative log of the probability of a pathogen being associated with a phenotype class *c*: *ic*(*c*) = −log *p*(*c*). Resnik’s similarity between two phenotype classes *c*_1_ and *c*_2_ is then defined as $sim({c}_{1},{c}_{2})={{\rm{\max }}}_{c\in S({c}_{1},{c}_{2})}ic(c)$ where $S({c}_{1},{c}_{2})=\{c| {c}_{1}\, \sqsubseteq \,c\,{\rm{and}}\,{c}_{2}\, \sqsubseteq \,c\}$, i.e., *S*(*c*_1_, *c*_2_) is the set of shared superclasses of *c*_1_ and *c*_2_. As pathogens are associated with multiple phenotypes and a phenotype-based query will also consist of multiple phenotypes, we use the Best-Match Average strategy to calculate the similarity between a pathogen *p* and a query *q* (both represented by sets of phenotypes):

1 $$si{m}_{BMA}(p,q)=\frac{\sum _{{c}_{1}\in p}\mathop{{\rm{\max }}\,}\limits_{{c}_{2}\in q}sim({c}_{1},{c}_{2})+\sum _{{c}_{1}\in q}\mathop{{\rm{\max }}\,}\limits_{{c}_{2}\in p}sim({c}_{1},{c}_{2})}{m+n}.$$

Additionally, we use the OPA2Vec framework^21^ to generate “ontology embeddings” for the pathogens, and we use a t-SNE dimensionality reduction^22^ to visualize the resulting embeddings and their relations. An ontology embedding is a function that maps classes, relations, and instances in an ontology to vectors within an n-dimensional vector space^21^.

### Implementation

We developed the web application for our database using Python Django framework (https://djangoproject.com/) for the backend and ReactJS (https://reactjs.org/) for the frontend services. We store only identifiers for the links in our database and retrieve all the annotations and additional data such as names from the AberOWL^23^ ontology repository using its REST API. For this purpose, we created compressed versions of DO, PhenomeNET and NCBITAXON^24^ ontologies.

## Data Records

PathoPhenoDB is available at http://patho.phenomebrowser.net and its content is accessible through a public SPARQL endpoint. Every release of the data is deposited in the Zenodo data repository^25^.

**Fig. 1:**
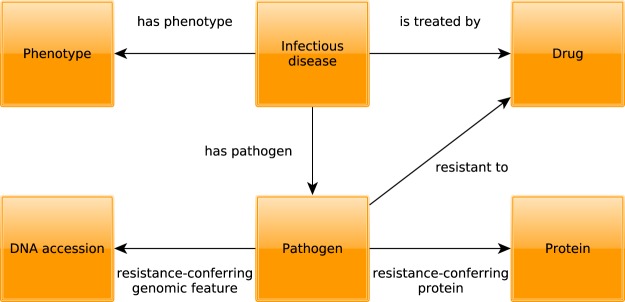
Schematic overview of the types of entities and their relations in PathoPhenoDB.

PathoPhenoDB is a database of human pathogens, the diseases and phenotypes they elicit in human organisms, and information related to drug treatments and mechanisms of drug resistance. Specifically, PathoPhenoDB contains associations between pathogens and diseases, between pathogens and phenotypes, between drugs that are approved to treat particular pathogens, and it identifies genes or proteins within pathogens that can convey resistance to particular drugs. Figure 1 provides a high-level overview of the information in PathoPhenoDB. PathoPhenoDB is constructed through a combination of manual curation of scientific literature, text mining, information extraction, and data integration approaches using Semantic Web technologies.

In PathoPhenoDB, we consider a pathogen to be any kind of bacterium, virus, fungus, protozoan, parasite, or other type of organism that is known to be able to cause a disease or abnormal phenotype in humans. With this broad view of pathogens, PathoPhenoDB includes 32 types of parasitic insects, 115 fungi, 208 bacteria, 47 protozoa, 175 viruses, and 115 taxa of parasitic worms. Our database currently covers a total of 1,170 pathogen–disease associations between 508 infectious diseases and 692 taxa of pathogens. For a total of 130 infectious diseases and 399 pathogen–disease associations, we also include information about drugs that can treat the disease and pathogen. We further include information on known mechanisms of drug resistance for 30 pathogens representing 78 pathogen–disease associations. While PathoPhenoDB is largely based on manually curated information, we also extracted pathogen–disease associations from the biomedical literature^9^ and use this information to enrich the content of our database. Statistics relevant to the text-mined content is available from the web site http://patho.phenomebrowser.net.

We use the Human Disease Ontology (DO)^13^ as a reference for infectious diseases in humans and base all our disease-related information on the DO. To associate pathogens with phenotypes, we follow a data integration approach and deductive inference where we utilize pathogen–disease associations to propagate phenotypes associated with infectious disease in DO^18^ to the pathogens that cause the diseases. We performed the inferences using the reasoner Elk, querying for subclass associations. The inferences of new subclass relations will be based on other axioms in the ontology. In PathoPhenoDB, we utilize 1,140 of 1,143 pathogen–disease associations to assign phenotypes for 476 (out of 508) infectious diseases to pathogens. For example, we use the phenotypes assigned to the DO class *Plasmodium malariae malaria* (DOID:14324), which includes phenotypes such as “episodic fever”, “hemolysis” and “anura”, and assign all phenotypes of this disease to *Plasmodium malariae* (NCBITaxon:5858) based on the association between *Plasmodium malariae* and *Plasmodium malariae malaria*.

As vocabulary for phenotypes we use a combination of the Human Phenotype Ontology (HPO)^16^ and the Mammalian Phenotype Ontology (MP)^26^. While both ontologies are formally distinct and use different identifiers, they can be integrated and aligned through cross-species phenotype ontologies such as PhenomeNET^6^ or UberPheno^27^. For our database, we use the PhenomeNET ontology as it has been applied in a variety of phenotype-driven studies of molecular mechanisms^28,29^. The 692 pathogens in PathoPhenoDB are associated with 1,719 distinct phenotypes from HPO and 479 distinct phenotypes from MP. On average, each pathogen is directly associated with 20 phenotypes.

Using ontologies to represent phenotypes enables deductive inferences using the ontology axioms^30,31^. To exploit these inferences, we represent the data in PathoPhenoDB using the RDF format (described in the methods section) and it is available for querying by using SPARQL endpoint from the web site. We use the Relation Ontology (RO)^32^ and the Semanticscience Integrated Ontology (SIO)^33^ to represent the relations between the entities. For the relations in Fig. 1, the has_phenotype relation corresponds to RO:0002200, has_pathogen to RO:0002556 and the is_treated_by relation to RO:0002302.

As we gathered pathogen–disease and disease–phenotype associations using two different methods – text mining and manual curation – we use the has_annotation relation from SIO (SIO:000255) to reify annotation assertions; in particular, we generate annotation objects that consists of a relation, an annotation value of the relation, and an evidence code that represents the level of evidence for the annotation. For example, to represent the information that *Actinomadura madurae* (NCBITaxon:1993) may cause *Actinomycosis* (DOID:8478), as obtained from text mining, we generate a new annotation object consisting of the *pathogen of* (RO:0002556) relation to *Actinomycosis* (DOID:8478) and the *has evidence* (RO:0002558) relation to *manual assertion* (ECO:0000203) in the Evidence and Conclusion Ontology (ECO)^34^.

In addition to reusing relations from established ontologies, we generate new relations (*resistant to*, *resistance-conferring protein*, *resistance-conferring genomic feature*) to capture information on drug resistance. We consider *resistant to* as a primitive relation between individuals: *x* is *resistant to y* if and only if *x* is a pathogen and *y* is a chemical compound, and *x* does not decrease its fitness when exposed to *y*. The class of pathogens *X* is resistant to the class of chemicals *Y* if and only if all instances of *X* are resistant to some instance of *Y*. Our intuition is that in order for *X* to be resistant to *Y*, *X* needs to contain a resistance-conferring feature, either encoded on DNA, RNA, or a protein. Being a resistance-conferring feature is a ternary relation that relates a class of pathogens, chemicals, and the resistance-conferring feature: *x* is a *resistance-conferring feature* for *y* with respect to *z* if and only if (a) *x* is a part of *y*, (b) *y* is resistant to *z*, (c) *x* is *normally* not a part of *y* and *y* is normally not resistant to *z*, and (d) *y* would not be resistant to *z* if *x* would not be a part of *y*. “Normally” here is a comparison to a reference or “normal” representation of the pathogen organism and which can be formalized, for example, through non-monotonic reasoning^35^.

We plan to update PathoPhenoDB regularly each year by running the text mining workflows. The data in PathoPhenoDB is also updated irregularly when new data becomes available or issues with existing data are resolved. To report issues such as incorrect or missing associations, we maintain an issue tracker. Data are released in RDF format and every release of the data is deposited in the Zenodo data repository^25^.

## Technical Validation

**Fig. 2:**
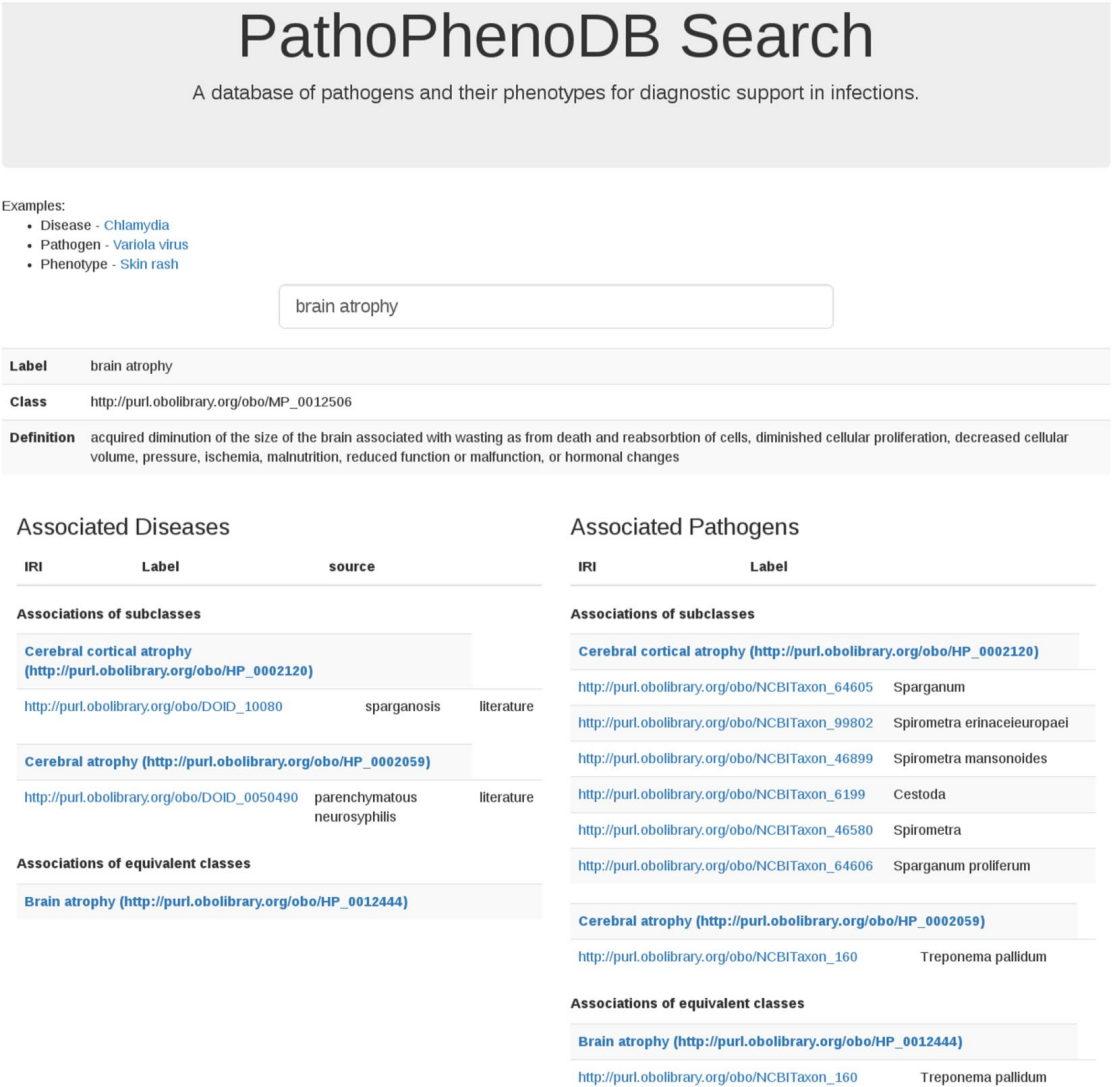
Search in PathoPhenoDB.

Figure 2 presents the search in PathoPhenoDB for a particular phenotype, *Brain atrophy*. The query retrieves all the infectious diseases associated with brain atrophy and their causative pathogens. Due to the use of inference and Semantic Web technologies, PathoPhenoDB can identify both direct and indirect associations between pathogens and phenotypes. We classify an association as direct if it is explicitly asserted during the curation. We classify an association as indirect if it is inferred based on a direct association and application of inference over the subclass hierarchy of the ontologies. For example, PathoPhenoDB does not cover any infectious disease that is directly associated with *Toxocara* (NCBITaxon:6264). However, a query for *Toxocara* will return all the disease and phenotype associations linked to its subclasses, including *Toxocara canis* (NCBITaxon:6265) and *Toxocara canti* (NCBITaxon:6266) as indirect associations. Using this kind of inference, PathoPhenoDB can provide useful and relevant knowledge on the entities of interest while using the background knowledge contained in the class hierarchy of the ontologies.

**Fig. 3:**
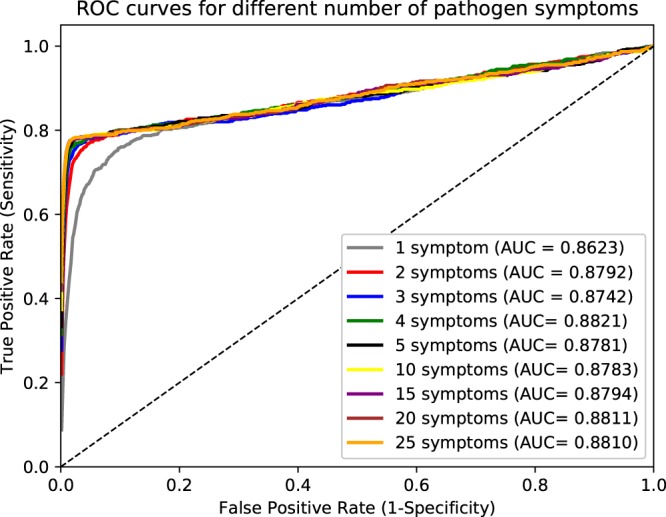
Pathogen recovery with different number of symptoms.

**Fig. 4:**
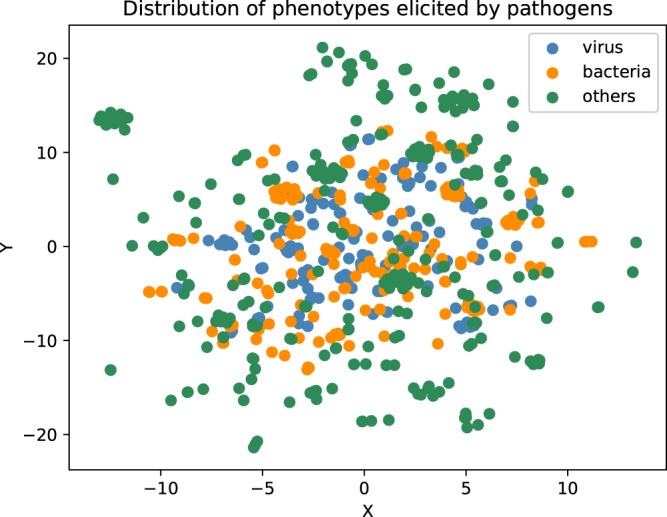
t-SNE plot of pathogens. Pathogens are represented using their ontology embeddings that have been generated using their associated phenotypes. Viruses are colored in blue, bacteria in orange, all other pathogens in green.

In addition to querying our database using Semantic Web technologies, the information in PathoPhenoDB can also be used to perform approximate queries using semantic similarity measures^20^. We test the similarity-based retrieval of pathogens by generating synthetic sets of phenotypes that consist of randomly chosen subsets of phenotypes associated with an infectious disease, and trying to identify the pathogen causing the disease based on semantic similarity over phenotype ontologies. Figure 3 shows the performance of recovering pathogens through semantic similarity when providing a varying number of symptoms. The model achieves a ROCAUC of over 86% using a single phenotype as query and does not significantly improve with more phenotypes added. We speculate that this is the result of pathogens falling in distinct phenotypic groups and that semantic similarity does not appropriately weight the distinguishing phenotypes within the group (because they are too general). We further use a data-driven semantic similarity measure based on ontology embeddings^21^ to visualize the pathogens and their disease phenotypes in a 2-dimensional space using a t-SNE dimensionality reduction^22^; phenotypically related pathogens are closer together in this space and the visualization of the embeddings can be used to identify pathogens that elicit similar phenotypes in their hosts. Figure 4 shows the resulting plot. We also make this figure available on our website to enable interactive exploration of pathogens based on their phenotype similarity.

Furthermore, the data in PathoPhenoDB has also been used to predict host-pathogen interactions^36^. The assumption in this application is that host phenotypes are the result of molecular interactions between the pathogen and a host protein. Comparing the phenotypes associated with pathogens in PathoPhenoDB and loss-of-function phenotypes could successfully predict these interactions^36^.

## Usage Notes

PathoPhenoDB covers information about known mechanisms of drug resistance (i.e., of genes and proteins that have the potential or disposition to convey resistance to certain drugs) gathered from the ARO^11^. This information can be used to determine (a) potential drugs to treat an infection caused by a particular pathogen, and (b) determine known mechanisms of resistance to such a treatment. It can be useful mainly in computational studies relying on data integration, but can also provide a link to the relevant resources that contain information about molecular markers of drug resistance.

One limitation of our dataset is that we do not handle variability of symptoms, mainly because we lack the necessary information to determine the frequency of symptoms in infections. For the most part, this information is not reported in the literature except as individual case studies. Instead, PathoPhenoDB contains a set of canonical symptoms and should be used in conjunction with a measure of relevance of a particular symptom such as through information content measures in semantic similarity computation.

Another limitation of our dataset is the inclusion of text mining results for some relations, specifically for pathogen–disease and disease–phenotype associations. We evaluate and report the performance of our text mining systems on these tasks, and while the majority of text mined relations will be accurate, there is nevertheless a probability that some information in PathoPhenoDB is incorrect. One source of low precision in our text mining evaluation is the use of ontologies and our ability to predict relations between more specific or more general types of entities, which we consider as false positive predictions. Furthermore, our text mining method has limitations due to its lexical components. When we or users of PathoPhenoDB identify incorrect or missing associations, we manually remove or add relations, and these manual additions can be identified from their evidence codes.

Infectious disease research and diagnosis of infectious disease is rapidly changing with the application of sequencing technologies. Current routine clinical pathogen identification methods often do not identify the most effective and specific treatment options^37^, or are not able to identify the causative pathogens rapidly. Recent achievements in next generation sequencing technologies (NGS) have led clinical microbiology to move in the direction of molecular diagnostic approaches^38^. NGS, in particular metagenomics and metatranscriptomics, can address the limitations of traditional microbial diagnostic methods by offering unbiased identification of organisms and can also be used to identify drug resistance and other functional information. Furthermore, metagenomics enables us to detect non-culturable organisms and multiple infections, and already shows great potential to be used in the rapid and accurate identification of pathogens^39,40^.

While NGS-based approaches have the potential to identify a wide range of pathogens in a single sequencing run, they may identify multiple different microorganisms that have the potential to cause infections. Identification of the causative pathogen among the set of pathogens identified through metagenomics approaches, is an additional challenge. In the future, matching the phenotypes observed in a patient to phenotype in PathoPhenoDB may provide additional features that can be combined with information from NGS to improve diagnosis and treatment of infectious disease.

As a more direct application of PathoPhenoDB, we envision its use in investigating molecular mechanisms underlying infectious diseases, specifically host-pathogen interactions. Phenotypes indirectly encode the molecular interactions between hosts and pathogens and therefore may be used to study the molecular basis of infectious disease^36^.

## ISA-Tab metadata file

Download metadata file

## Data Availability

The source code for PathoPhenoDB is freely available at https://github.com/bio-ontology-research-group/pathophenodb.
